# Productivity losses among individuals with common mental illness and comorbid cardiovascular disease in rural Karnataka, India

**DOI:** 10.4103/jncd.jncd_17_19

**Published:** 2019-09-27

**Authors:** Farah Naaz Fathima, James G Kahn, Srinivasan Krishnamachari, Maria Ekstrand

**Affiliations:** 1 Department of Community Health, St. John’s Medical College, Bengaluru, Karnataka, India; 2 Department of Epidemiology and Biostatistics, Global Health Sciences, Philip R. Lee Institute for Health Policy Studies, University of California, San Francisco, California, USA; 3 Department of Psychiatry, St. John’s Medical College, Bengaluru, Karnataka, India; 4 Department of Medicine, University of California, San Francisco, California, USA

**Keywords:** Cardiovascular disease, common mental illness, productivity losses

## Abstract

**Context:**

Common mental disorders (CMD) and cardiovascular diseases (CVDs) frequently co-occur. Productivity losses due to these diseases are substantial in high-income countries. Similar data from the developing world are lacking.

**Aims:**

This study aims to quantify productivity losses among individuals with comorbid CMD and CVD in rural Karnataka, India.

**Settings and Design:**

A cross-sectional study was done among patients with a dual diagnosis of a comorbid CMD and CVD in a district in Karnataka, India.

**Subjects and Methods:**

Three hundred and three patients were administered the iMTA Productivity Cost Questionnaire to measure losses of productivity at paid work (absenteeism and presenteeism) and unpaid work.

**Statistical Analysis Used:**

Valuation of productivity losses was done by multiplying the number of days of lost productivity by the standard value of productivity based on the minimum wage for agricultural work.

**Results:**

Among individuals with dual CMD and CVD, 76% had productivity losses. These losses were higher at unpaid (62%) than at paid work (32%). At paid work, losses due to presenteeism were greater than those due to absenteeism. The total days of productivity loss were 1204, amounting to 14.2% of the available person-days. The total productivity loss among 303 individuals with mental illness and comorbid CVD over a 4-week period amounted to 30.3 INR (0.47 USD) per person per day, representing 9.9% of total potential productivity.

**Conclusions:**

Productivity losses due to common mental illnesses and CVDs are high. There is a need to conduct more studies in this field.

## Introduction

Noncommunicable diseases (NCDs) are a major health and development challenges of the 21^st^ century. NCDs were responsible for 39.5 million (70%) of the world’s 56.4 million deaths in 2015. Cardiovascular diseases (CVD) were responsible for 45% of all NCD deaths.^[1]^ In India, NCDs were responsible for 5.87 million deaths, accounting for 60% of all deaths in 2014.^[2]^ The Global Burden of Disease study reports mental and substance abuse disorders as the second leading cause of disability worldwide, accounting for approximately 21% of global disability burden. Major depressive disorder was a crucial contributor in developed and developing countries alike: it is the leading cause of years lived with disease in 56 countries including India, the second leading cause in 56 countries, and the third in 34 countries.^[3]^

Mental disorders, especially depression, frequently co-occur with CVD. About 22%–33% of patients with CVD have clinical depression.^[4]^ Results from studies done in tertiary care centers showed that about 23%–25% of patients with CVD had a diagnosable psychiatric syndrome.^[5,6]^ The relationship between CVD and mental illness is bidirectional patients with CVD are at greater risk for depression, and patients with mental disorders are at greater risk for certain CVDs. Poor mental health can be a biological precursor to or a consequence of NCDs, and it can exacerbate NCD behavioral risk factors such as unhealthy diet, physical inactivity, tobacco use, and excess alcohol use.^[7]^ Individuals with depression are less likely to adhere to prescribed medication and health-promoting practices. Further, individuals with mental illness are likely to receive less care in nonmental health treatment settings.^[4]^ A cohort study done in Denmark, Finland, and Sweden report that individuals with mental disorders live 8–20 years fewer than the general population and usually die from causes such as NCDs (CVD, cancer, and pulmonary disease). The causes of death among patients with mental illness are similar to the causes of death in the general population.^[8]^

Patients suffering from mental illness have productivity losses due to absenteeism and presenteeism. While absenteeism means the absence of a worker due to illness, presenteeism refers to working less productively when one is ill.^[9]^

Mitchell and Bates studied a large sample of health risk appraisal data, quantifying the productivity impact due to absenteeism and presenteeism associated with various health conditions. They found that depression ranked third among health conditions with an annual productivity loss per person of 878 USD. The annual productivity cost per person for CVDs, including heart disease, diabetes mellitus, blood pressure, and obesity were 328, 324, 230, and 203 USD, respectively.^[10]^ Results from a study by Lim *et al.,* from Australia shows that full-time workers with mental disorders lost an average of 1 day due to work loss (absenteeism) in the past 1 month along with an average of 3 days due to presenteeism.^[11]^

Available data on productivity losses are mostly from high-income countries.^[12,13]^ A systematic review on the productivity losses associated with CVD identified a total of 60 studies, including 20 from the USA, 25 from Europe, and 18 from other countries (Australia, Canada, Indonesia, and Korea). The annual productivity losses reported for morbidity and mortality from the United States were USD 11 billion, 41 billion, and 192 billion for heart failure, stroke, and all CVD, respectively. The annual presenteeism costs per employee were USD 288 for CHD and USD 5,128 for CHD or stroke. Studies from Europe show that total annual population productivity losses due to CVD are €4.9 billion (US$ 5.4 billion) from premature mortality and €120 million (US$133 million) from morbidity.^[14]^

The results of the National Mental Health Survey in India^[15]^ show that two of three individuals with depression reported disability in work (67.3%), family (70.2%), and social life (68.6%). Nearly 21% of affected individuals reported substantial difficulties in carrying out daily activities, for an average of 20 days in the previous month. An Indian family spends around Rs. 1500 (23 USD) per month toward care of individuals with mental illness. This includes cost toward consultation, drugs, and transport.^[15]^

The report of economics of NCDs in India published by the Harvard School of Public Health states that loss to Indian economy due to NCDs and mental health conditions between 2012 and 2030 is 4.58 trillion USD, out of which CVDs account for 2.17 trillion USD, and mental health conditions for another 1.03 trillion USD.^[16]^

The objective of this paper is to quantify productivity losses among individuals with common mental illness and comorbid CVD in rural Karnataka, India.

## Subjects and Methods

In this paper, we present the baseline results of a 3-month economic substudy funded through a training fellowship grant through the first author.^[17]^ The individuals for this analysis were recruited from an ongoing randomized trial (HOPE) set in rural South India. HOPE includes 2500 participants from 50 Primary Health Centers (PHC) in Ramanagaram District, Karnataka state.^[18]^

Participants in HOPE were adults (≥30 years) with a diagnosis of a common mental illness (Depression or Anxiety Disorder) and at least one CVD (hypertension, diabetes, hyperlipidemia, or ischemic heart disease) who were recruited using a two-step screening procedure. The initial screening consisted of an assessment of mental competency and psychological distress, self-reported diagnosis of a CVD using Kessler-10 score ≥6,^[19]^ capillary blood sugar ≥160 mg/dL, blood pressure ≥140/90 mmHg, possible angina by the Rose angina questionnaire^[20]^ and self-reported physician-diagnosed history of DM, hypertension, or ischemic heart disease. Any person who had a positive result on any of above tests was referred to the PHC-based study staff with a study card for the confirmatory screening of common mental illness using the Mini-International Neuropsychiatric Interview (MINI)^[21]^ and physician-diagnosed CVD, or one or more confirmed laboratory test. Participants who scored in at least the mild depression range were referred to the medical officer at the PHC and for treatment as appropriate, based on the collaborative care model.

Suicidal ideation was assessed following an affirmative answer to the PHC “trigger question,” using items from the MINI and participants at high suicidal risk were referred to the district psychiatrist for management and treatment. Baseline assessment of common mental illness was done using Generalized Anxiety Disorder Scale (GAD-7),^[22]^ and Patient Health Questionnaire Depression Scale (PHQ-9)^[23]^ was used to assess the severity of anxiety and depression. Validated local language translations of all the tools were used. PHQ 9 has good internal consistency (Cronbach’s alpha 0.89) and inter-rater reliability (intraclass correlation coefficient, 0.94). For a cutoff score of ≥9, PHQ-9 has a sensitivity of 82.5%, and a specificity of 90.1%.^[24]^ GAD 7 has good reliability and validity with a sensitivity of 89% and a specificity of 82%.^[25]^

The details of the screening tests and the cutoffs used are described elsewhere.^[18]^

For the current substudy, 303 participants were recruited consecutively over a period of 5 months during the year 3 of the present study. Written informed consent was sought from the participants.

We used the iMTA Productivity Cost Questionnaire (iPCQ) developed by the Institute for Medical Technology Assessment, the Netherlands. The iPCQ is a generic questionnaire designed to determine illness-related productivity losses was administered. iPCQ is a feasible and reliable instrument for collecting data on medical consumption and productivity losses in patients with mild-to-moderate mental health problems.^[26,27]^

In the iPCQ, absenteeism is quantified by multiplying the number of days missed by the number of hours per workday of the respondent. Presenteeism is calculated in terms of a number of workdays on which the respondent’s performance was hindered by health problems, and the respondent’s estimate of the amount of work he/she could perform on such days, compared with a fully functional workday. This estimate is expressed as an efficiency score ranging from 0 (present at work but unable to function) to 10 (bothered, but able to do as much as during a normal workday. The formula for calculating the number of hours of lost productivity due to “presenteeism” was as follows: number of workdays impaired × (1 − [efficiency score/10]) × number of hours per workday. Unpaid productivity losses were determined by multiplying the number of days of unpaid work missed by the number of hours of help needed per day to make up the work.

Valuation of productivity losses was done by multiplying the number of days of lost productivity by the standard value of productivity which was based on the minimum wage for agricultural work in Karnataka state as published by the Labor Department of Government of Karnataka.^[28]^ The cost of unpaid productivity loss was calculated similarly since a standard hourly rate for household care was not available.

Permission to use the tool was obtained from iMTA. There are no published studies using iPCQ in India. We translated (forward and backward) the tool into the local language (Kannada). The aim of translation was to produce an easy and natural-sounding translation that is acceptable to respondents in the target language and ensuring semantic equivalence.^[29]^

### Data analytic procedures

Data management was done using an Epi Info database (version 7, Center for Disease Control and Prevention, Atlanta, Georgia, USA). Variable listing and coding of the questionnaires were done by the statistical team. This process was checked by the first author. Issues and corrections were discussed with the data management team. A random sample of 10% of the entries was cross-checked by the investigator.

Data were analyzed using STATA version 14 (StataCorp LLC, Texas, USA). Sociodemographic characteristics of the study population and the proportion reporting productivity losses were quantified using descriptive statistics such as frequencies, percentages, means, and standard deviation.

Productivity losses were calculated using the methods described in the PCQ Manual by Productivity and Health Research Group from Institute for Medical Technology Assessment.^[26,27]^

The standard daily minimum wage for Karnataka state as per the labor department of the Government of Karnataka is 304 Indian National Rupees for 2015–2016.^[28]^ This was considered as the standard cost price of productivity per day for our analysis.

The total cost of productivity loss was calculated by summing the productivity losses due to presenteeism and absenteeism at work, and for unpaid work.

The association between the proportion of participants reporting productivity losses with age and gender was studied using the Chi-square test for association and a *P* < 0.05 was considered statistically significant for all analyses.

## Results

The mean age of the study population was 59.9 ± 9.1 years with 76.9% females and 64.4% elderly (≥60 years). A large proportion (67.3%) had not attended school. One-third of the individuals (33.7%) had a paid job, 22.7% were homemakers, and 42.6% were retired. The age and gender distribution of the study population is depicted in Table 1.

The study participants in the parent study were adults (≥30 years) and had a diagnosis of common mental illness and at least one CVD. Table 2 describes the morbidity profile of the study participants.

**Figure 1:**
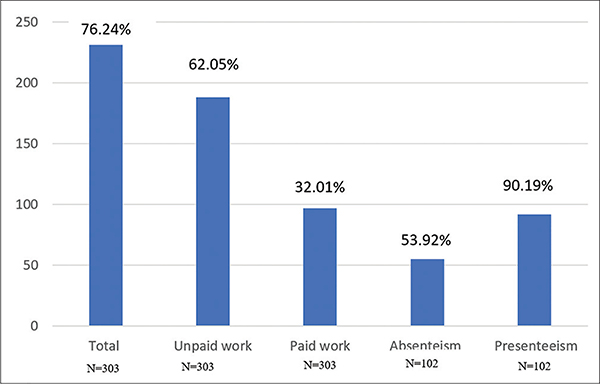
Proportion of study participants with productivity losses. *N* = 303 for “Total,” “Unpaid work” and “Paid work.” *N* = 102 for “Absenteism” and “Presenteeism”

Figure 1 depicts the proportion of study participants with productivity losses. Of 303 participants, 231 (76%) reported some form of productivity losses in paid and/or unpaid work. Most of these individuals reported productivity loss at unpaid work (188%, 62%) whereas 32% (97 individuals) reported productivity loss at paid work. Out of 102 individuals who had a paid job, 55 (53.9%) reported productivity loss due to absenteeism and 92 (90.2%) due to presenteeism.

The mean number of days of missed work in the past 4 weeks among the 55 individuals who reported absenteeism from paid work was 7.5 ± 5.1 days. The mean number of days worked despite illness among the 92 individuals who reported presenteeism at paid work in the past 4 weeks was 6.9 ± 6.4 days.

A total of 8484 productive person-days were available for 303 individuals over the prior 4 weeks. The total productivity loss was 1204 days, amounting to 14.2% of the available person-days. Out of this, 412 (34.2%) productive days were lost due to absenteeism from paid work; 449 days (37.3%) were lost due to presenteeism at paid work, and 343 days (28.5%) due to productivity loss at unpaid work.

The standard minimum wage for Karnataka state as per the labor department of the Government of Karnataka for 2015–2016 is 304 Indian National Rupees (4.68 USD) per day. Therefore, the total productivity loss among 303 individuals with mental illness and comorbid CVD over a 4-week period was 256,697 INR (3,949 USD). This amounts to 30.3 INR (0.47 USD) per person per day, representing 9.9% of the total potential productivity.

The total productivity losses at unpaid work among the 303 study participants total 72,249 INR (1111 USD) whereas the total productivity losses at paid work were 184,448 INR (2837 USD) out of which absenteeism contributed to 89,604 INR (1378 INR) and presenteeism at work contributed to 94,844 INR (1536 USD).

**Figure 2:**
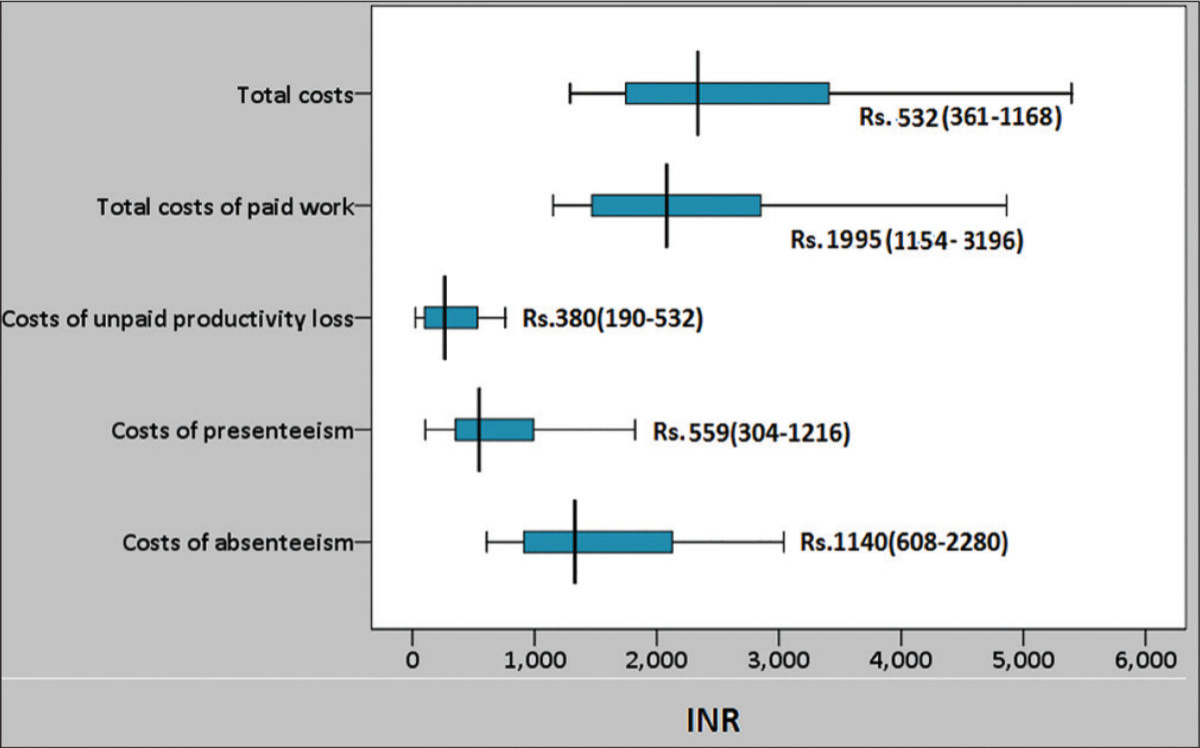
Median cost of productivity losses. Values depicted in the graph are median and IQR. All cost are depicted in Indian Rupees. *N* = 303 for “Total,” “Unpaid work” and “Paid work.” *N* = 102 for “Absenteism” and “Presenteeism”

Figure 2 depicts the median costs of productivity losses. The median cost of productivity loss due to paid work was 1995 (IQR 1154-–196), and the median cost of productivity loss due to absenteeism was 1140 (IQR 608–2280). There was no difference in the median costs of productivity losses between those who had individual psychiatric morbidity and multiple morbidities.

Our results showed that 92.9% of the males and 72.9% of the females reported total productivity losses (*P* < 0.001). Furthermore, 80% of the males and 56.2% of the females reported productivity losses at unpaid work (*P* < 0.001). Detailed productivity losses by age and gender are depicted in Table 3.

## Discussion

This study found that a large majority (76%) of individuals with a common mental illness and comorbid CVD had productivity losses. Productivity losses are common at unpaid (58%) and paid work (32%). These costs are depicted in Table 4. Productivity losses due to presenteeism (27.5%) at work were greater than those due to absenteeism (16.4%).

The National Mental Health Survey in India (2015–2016) showed that every two of three individuals with depression reported disability in work life (67.3%), family life (70.2%), and social life (68.6%). Nearly 21% of affected individuals reported substantial difficulties in carrying out their daily activities. The results of our study corroborate the high impact of common mental disorders on productivity in individuals with mental illness in India.^[15]^

The results of the National Mental Health Survey in India also report that an Indian family spends around 1500 INR (23 USD) per month toward consultation, drugs, and transport for care of individuals with mental illness. This is high, considering the fact that the annual median per capita income in India (2014) is USD 616 (USD 51.3 per month).^[30]^ The Total Health Expenditure as a % of Gross Domestic Product is 4.02% with a per capita estimate of INR 3638.3 suggesting that around half the household health expenditure in India is on mental illnesses.^[31]^ In addition to the direct costs quantified by the National Mental Health Survey, our study quantifies indirect costs due to productivity losses among individuals with mental illness and a comorbid CVD. In our study, the total indirect costs in mental illness amounted to 900 INR per person per month, which is more than one-third of the total costs in mental illness.

Our results showed that productivity losses for unpaid work are high (56% of individuals and 36.5% of days). These are often neglected and unaccounted for while costing and may lead to underestimation of the problem by policymakers.

The productivity losses observed were much higher than for a general population. Mitchell and Bates^[10]^ reported that the mean annual absent days among individuals without any underlying health problem to be 1.4 and mean annual unproductive days among those with health problems to be 3.7. Persons with two or more morbidities missed an average of 3.6 days of work in a year and were unproductive on an average of 20.1 days. Our study shows that the mean annual absent days among individuals with mental illness and comorbid CVD/DM to be much higher at 89.8 days/year and the mean annual unproductive days to be 82.4 days per year.

The average productivity loss in our study population was 30.26 INR (0.47 USD) per capita per day, which is high for an Indian setting where 21.2% of population live below the world bank poverty line definition of 1.9 USD per day. In the Indian context, the Rangarajan Committee set up by the Reserve Bank of India in 2014 defines poverty line as less than INR 32 per capita per day for rural areas and INR 47 per capita per day in urban areas.^[30]^ Based on this estimate, a productivity loss of 30.26 INR per capita per head for mental illness and comorbid CVD is high.

Studies have shown that mood and anxiety disorders are more common in females.^[32,33]^ We found that productivity losses are higher among males, for total productivity loss and for losses at unpaid work. The gender difference in total productivity loss could be partly because a higher proportion of males (42.9%) had a paid job than females (30.9%). In addition, the mean age of the male participants (63.83 ± 10.16 years) was higher than that for females (58.73 ± 9.54 years). The higher age of the male participants could be because participants for the parent study were recruited from PHC on working days when many younger males would be at work. In addition, the younger males may not have consented to participate in the parent study as it would require multiple visits to the health-care center. The overall ages of both male and female participants in our study were high which could be explained by the inclusion criteria of the parent study wherein participants had to have a common mental illness and at least one comorbid CVD.

Males also reported higher productivity losses at unpaid work which could be explained by the fact that 27% of the females being housewives could not afford to miss unpaid household work despite illness. Males in rural India, on the other hand, traditionally do not contribute to domestic household activities and the productivity losses due to unpaid work could have been due to leisure time activities which are easier to miss for males.

Our study has some limitations. First, since all our study participants had common mental illness and comorbid CVD/diabetes, it was impossible to separate the contribution to productivity losses due to each of these conditions. Second, the estimation of days of productivity losses was by self-report, and thus subject to recall errors. The quantification of productivity losses due to presenteeism was subjective since the participants were asked to estimate the efficiency of work performance on days when they worked despite being unwell.

Despite these limitations, our study provides important information on the magnitude of productivity losses due to common mental illness and comorbid CVD. To the best of our knowledge, this is the first study that quantifies the productivity losses among individuals with mental illness and a comorbid CVD in a rural Indian setting. Many developed countries make policy decisions based in part on the cost-effectiveness of interventions. Similar data from India are lacking. Studies like ours that look at baseline productivity losses and subsequently, at the changes in productivity following an intervention can have policy implications in terms of identification of interventions that improve productivity.

## Conclusions

Our study shows that productivity losses due to common mental illnesses and cardiovascular diseases are high in a rural Indian setting. There is a need to conduct more studies in this field to estimate productivity losses in Indian settings.

## Figures and Tables

**Table 1:** Age and gender distribution of the study population

Age category (years)	Male, *n* (%)	Females, *n* (%)	Total

<60	15(13.3)	98 (86.7)	113
≥60	55 (28.9)	135 (71.0)	190
Total	70 (22.7)	233 (75.9)	303

**Table 2:** Morbidity profile of the study participants

Morbidity	*n* (%)

At least moderate anxiety with generalized anxiety disorder 7	86 (28.7)
score ≥10	
At least moderate depression PHQ 9 score ≥ 10	123 (41.0)
BMI ≥25 kg/m^2^	125 (41.7)
Blood pressure ≥140/90 mmHg	84 (28.0)
HbAlc ≥6.5	182 (60.7)
Total cholesterol ≥200 mg/dl	139 (44.0)

PHQ - Patient health questionnaire, BMI - Body mass index, HbA1c - Hemoglobin A1C

**Table 3:** Proportion of the study participants reporting productivity losses by age and gender

Variables	Total (*n*=231)	Unpaid work (*n*=188)	All paid work (*n*=97)	Absenteeism from work (*n*=55)	Presenteeismat work (*n*=92)

Gender					
Male (*n*=70)	65 (92.9)	56 (80)	29 (41.4)	13(43.3)	28 (93.3)
Female (*n*=223)	166 (72.9)	132(56.7)	68 (29.2)	42 (58.3)	64 (88.9)
P value for gender difference	<0.001^a^	<0.001^b^	0.06^b^	0.19^b^	0.71^b^
Age (years)					
30−39 (*n*=10)	7(70)	6(60)	6(60)	5 (50.0)	6(60)
40−49 (*n*=40)	33 (82.5)	27 (67.5)	13(32.5)	9 (22.5)	13(32.5)
50−59 (*n*=63)	39 (61.9)	33 (52.4)	18(28.6)	14(22.2)	17(27)
≥60 (*n*=190)	152 (80)	122 (64.2)	60 (31.6)	27(14.2)	56 (29.5)
P value for age difference	0.02^b^	0.29^b^	0.28^b^	0.02^b^	0.22^b^

a Chi square test,

b Fisher's exact test. Figures in parenthesis indicate row percentages

**Table 4:** Total cost of productivity loss in INR over 4 weeks among 303 participants

Type of productivity loss	Cost (in INR)
Total	2.56,697
Unpaid work	72.249
Paid work	1,84,448
Absenteeism	89,604
Presenteeism	94,844
